# Diatoms Are Selective Segregators in Global Ocean Planktonic Communities

**DOI:** 10.1128/mSystems.00444-19

**Published:** 2020-01-21

**Authors:** Flora Vincent, Chris Bowler

**Affiliations:** a lnstitut de Biologie de l’ENS, Département de Biologie, École Normale Supérieure, CNRS, INSERM, Université PSL, Paris, France; b Research Federation for the Study of Global Ocean Systems Ecology and Evolution, FR2022/Tara Oceans GOSEE, Paris, France; University of Vienna

**Keywords:** cooccurrence networks, environmental microbiology, marine microbiology, phytoplankton, protists

## Abstract

Diatoms are a major component of phytoplankton, believed to be responsible for around 20% of the annual primary production on Earth. As abundant and ubiquitous organisms, they are known to establish biotic interactions with many other members of plankton. Through analyses of cooccurrence networks derived from the *Tara* Oceans expedition that take into account both biotic and abiotic factors in shaping the spatial distributions of species, we show that only 13% of diatom pairwise associations are driven by environmental conditions; the vast majority are independent of abiotic factors. In contrast to most other plankton groups, on a global scale, diatoms display a much higher proportion of negative correlations with other organisms, particularly toward potential predators and parasites, suggesting that their biogeography is constrained by top-down pressure. Genus-level analyses indicate that abundant diatoms are not necessarily the most connected and that species-specific abundance distribution patterns lead to negative associations with other organisms. In order to move forward in the biological interpretation of cooccurrence networks, an open-access extensive literature survey of diatom biotic interactions was compiled, of which 18.5% were recovered in the computed network. This result reveals the extent of what likely remains to be discovered in the field of planktonic biotic interactions, even for one of the best-known organismal groups.

**IMPORTANCE** Diatoms are key phytoplankton in the modern ocean that are involved in numerous biotic interactions, ranging from symbiosis to predation and viral infection, which have considerable effects on global biogeochemical cycles. However, despite recent large-scale studies of plankton, we are still lacking a comprehensive picture of the diversity of diatom biotic interactions in the marine microbial community. Through the ecological interpretation of both inferred microbial association networks and available knowledge on diatom interactions compiled in an open-access database, we propose an ecosystems approach for exploring diatom interactions in the ocean.

## Abstract (precis)

Diatoms are key phytoplankton in the modern ocean that are involved in numerous biotic interactions, ranging from symbiosis to predation and viral infection, which have considerable effects on global biogeochemical cycles. However, despite recent large-scale studies of plankton, we are still lacking a comprehensive picture of the diversity of diatom biotic interactions in the marine microbial community. Through the ecological interpretation of both inferred microbial association networks and available knowledge on diatom interactions compiled in an open-access database, we propose an ecosystems approach for exploring diatom interactions in the ocean.

## INTRODUCTION

Marine microbial communities, composed of bacteria, archaea, and protists, as well as viruses, play essential roles in the functioning and regulation of Earth’s biogeochemical cycles (1). Their roles within planktonic ecosystems have typically been studied under the prism of bottom-up research, namely, understanding how resources and abiotic factors affect their abundance, diversity, and functions. On the other hand, the effects of mortality, allelopathy, symbiosis, and other biotic processes are also likely to shape their communities and to exert strong selective pressures, yet they have been studied much less. With concentrations reaching 10^7^ protists (2) and 10^9^ prokaryotes (3, 4) per liter of sea water, biotic interactions are likely to impact community structure from the microscale to the ecosystem level (5).

Among marine protists, diatoms (Bacillariophyta) are of key ecological importance. They are a ubiquitous and predominant component of phytoplankton, characterized by their ornate silica cell walls, and are considered to be responsible for approximately 40% of marine net primary productivity (NPP) (6, 7). The array of biotic interactions in which marine diatoms have been described is vast. They are fed upon by heterotrophic microzooplankton such as ciliates and phagotrophic dinoflagellates (8–10) as well as by metazoan grazers such as copepods (11–14). Other known interactions include symbioses with nitrogen-fixing cyanobacteria (15, 16) and tintinnids (17), parasitism by chytrids and diplonemids (18), diatom-targeted allelopathy by algicidal prokaryotes and dinoflagellates (19, 20), and allelopathy mediated by diatom-derived compounds detrimental to copepod growth (21, 22). Beyond direct biotic interactions, diatoms are also known to thrive in high-nutrient and high-turbulence environments, such as upwelling regions, at the expense of the other major phytoplankton groups, for instance, dinoflagellates and haptophytes (23, 24). Competition for silicon between diatoms and radiolarians, other silicifying members of plankton, has also been noted (25, 26).

Diatoms are one of the most diverse planktonic groups in terms of species, widely distributed across the world’s sunlit ocean (27) and capable of generating massive “blooms” in which the diatom biomass can increase up to 3 orders of magnitude in just a few days (28). Their success has been attributed, in part, to a broad range of predation avoidance mechanisms (29), such as their solid mineral skeleton (30), chain and spine formation in some species, and toxic aldehyde production (31, 32). However, a global view of their capacity to interact with other organisms and an assessment of the impact of diatom interactions on community composition are still lacking.

Cooccurrence networks using meta-omics data are increasingly being used to study microbial communities and interactions (33, 34), e.g., in human and soil microbiomes (35, 36) as well as in marine and lake bacterioplankton (37–39). Such networks provide an opportunity to extend community analysis beyond alpha and beta diversity toward a simulated representation of the relational roles played by different organisms, many of which are uncultured and uncharacterized (40, 41). Over large spatial scales, nonrandom patterns according to which organisms frequently or never occur in the same samples are the result of several processes, such as biotic interactions, habitat filtering, as well as neutral processes (42). Quantifying the relative importance of each component is still in its infancy. However, these networks can be used to reveal niche spaces, to identify potential biotic interactions, and to guide more focused studies. Much like in protein-protein networks, interpreting microbial association networks also relies on literature-curated gold-standard databases (34), although such references are woefully incomplete for most planktonic groups (43).

As part of the recent *Tara* Oceans expedition (44, 45), determinants of community structure in global ocean plankton communities were assessed using cooccurrence networks (46), based on the abundances of viruses, bacteria, metazoans, and protists across 68 *Tara* Oceans stations in two depth layers in the photic zone. Pairwise links between species were computed based on how frequently they were found to cooccur in similar samples (positive correlations; here named copresences) or, on the contrary, if the presence of one organism negatively correlated with the presence of another (negative correlations; here named exclusions). It should be noted that our use here of the terms copresence and exclusion does not imply any type of biotic interaction or active process from either of the partners. In order to prevent spurious correlations due to the presence of additional confounding components such as abiotic factors, interaction information was furthermore calculated to assess whether or not correlations were driven by an environmental parameter. The *Tara* Oceans interactome has global coverage and reports over 90,000 statistically significant correlations, with ∼68,000 of them being positive, ∼26,000 of them being negative, and ∼9,000 being due to the simultaneous higher correlation of two organisms (operational taxonomic units [OTUs]) with a third environmental parameter.

In this study, we provide an in-depth analysis of the diatom interactome in the open ocean, involving both prokaryotic and eukaryotic partners. We show how species distribution patterns reveal segregation between diatoms and specific taxonomic groups. We further investigate network properties involving the groups with which diatoms display the highest numbers of associations and reveal ecologically relevant areas of potential research by comparing the diatom interactome with literature previously published on the topic.

## RESULTS

### Diatoms are segregators in the open ocean.

In the *Tara* Oceans interactome, we found diatoms to be involved in 4,369 interactions, making them the 7th most connected taxonomic group after syndiniales (marine alveolates [MALVs]), arthropods, dinophyceae, polycystines, marine stramenopiles (MAST), and prymnesiophyceae, independently from the taxon’s abundance (46). Overall, diatoms represent around 3% of all copresences (2,120/68,856) and 9.5% of all exclusions (2,249/23,777), showing that their contribution to exclusions is much greater than their contribution to copresences, contrasting with all the other major taxonomic groups in the interactome. The positive-to-negative ratio of the number of associations provides a measure of the group’s role in the network on a global scale. Diatoms (ratio = 0.99) and polycystines (ratio = 0.66) are the only two groups that have more negative than positive associations and thus can be defined as “segregators” following the definition of Morueta-Holme et al. (47), a term that does not imply any active mechanism but rather describes an abundance pattern (see Table S1 in the supplemental material).

10.1128/mSystems.00444-19.7 TABLE S1 Principal statistics of major taxonomic groups in the *Tara* Oceans interactome. Based on the *Tara* Oceans interactome, the taxonomic groups involved in the highest numbers of interactions were identified. For each group, the relative proportion of positive to negative interactions has been computed. Download Table S1, XLSX file, 0.01 MB. Copyright © 2020 Vincent and Bowler. 2020 Vincent and Bowler This content is distributed under the terms of the Creative Commons Attribution 4.0 International license.

**FIG 1:**
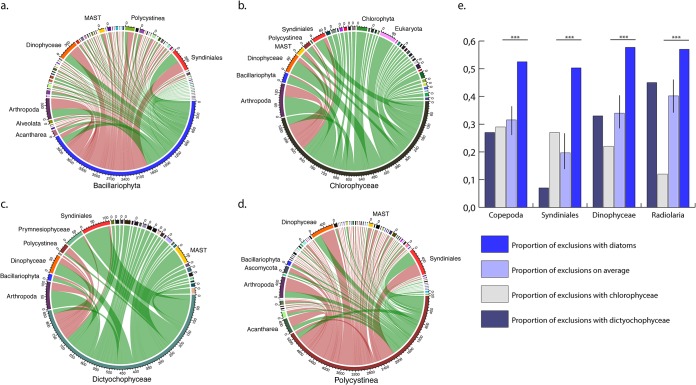
Major patterns of interactions for diatoms and control groups. (a to d) Circular representation of copresences (green bands) and exclusions (red bands) within subnetworks extracted from the *Tara* Oceans interactome (46) for diatoms (a), chlorophyceae (green alga control group) (b), dictyochophyceae (silicifying biflagellate mixotrophs) (c), and polycystines (the only other segregator) (d), with other taxa. The thickness of the band corresponds to the number of interactions, and major partners are labeled around the circles if they represent more than 100 associations. Data from all size fraction networks are represented here. (e) Comparison of proportions of exclusions showing that diatoms significantly exclude potential predators, parasites, and competitors such as copepods, Syndiniales, Dinophyceae, and Radiolarians, compared to control groups.

**FIG 2:**
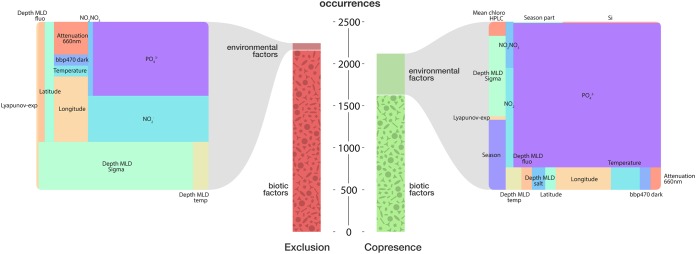
Biotic versus abiotic drivers of diatom interactions. The central scale shows the number of diatom copresences and exclusions that are best explained by biotic (bottom) and abiotic (top) factors. The left and right panels are closeup views of the abiotic drivers, where the size of the rectangles is relative to the number of correlations that are best explained by each parameter. Abbreviations: PO_4_^3−^, phosphate; MLD, mixed-layer depth (layer in which active turbulence homogenizes water, estimated by density [sigma] and temperature); NO_2_^−^, nitrite; Attenuation 660nm, light scattering by suspended particles; bbp470, backscattering coefficient of particles; HPLC chloro, chlorophyll pigment measurement (high-performance liquid chromatography adjusted); Lyapunov-exp, ocean perturbation (Lyapunov exponent); Si, silicate; Season, categorical variable for season. A full description of the environmental parameters is available on the PANGAEA website (https://doi.pangaea.de/10.1594/PANGAEA.840718).

A finer analysis revealed the major taxonomic groups with which diatoms correlate or anticorrelate. Copresences involve mainly arthropoda (9.2% of diatom copresences), dinophyceae (8.7%), and syndiniales (an order of dinoflagellates, also known as MALVs, found as parasites of crustaceans, protists, and fish) (11.7%). Exclusions include the three above-described groups, arthropoda (11.5%), dinophyceae (11.3%), and syndiniales (11.1%), as well as the polycystina (6%), a major group of radiolarians that produce mineral skeletons made from silica (Fig. 1a). Chlorophyceae were used as a control class for obligate photosynthetic green algae, and dictyochophyceae were used as a control class for silicified phytoplankton: both photosynthetic classes show more copresences with the above-mentioned groups (Fig. 1b and c). However, polycystines show exclusion trends similar to those of diatoms (Fig. 1d). The number of exclusions involving diatoms with arthropods, dinophyceae, syndiniales, and polycystines was much higher than what would be expected at random based on binomial testing (Fig. 1e), a pattern that was not found in other phytoplankton control groups. However, polycystines also display more exclusions than what would be expected at random with copepods, syndiniales, and dinoflagellates (Table S2). Among all the pairwise associations involving diatoms and other organisms in plankton (*n* = 4,369), only 13% were due to a third environmental parameter, suggesting a shared preference for a particular abiotic condition (*n* = 566). Therefore, 87% of the associations are best explained by the abundance of the two organisms alone (Fig. 2). Polycystines displayed a similar pattern, with 95% of the associations explained by biotic rather than abiotic interactions.

10.1128/mSystems.00444-19.8 TABLE S2 Binomial testing for diatoms and control groups. The likelihood of diatoms displaying negative interactions with the groups with which they interact the most is evaluated by binomial testing. Tests are also performed for control groups of Radiolaria, Dictyochophyceae, and Chlorophyceae. Download Table S2, XLSX file, 0.01 MB. Copyright © 2020 Vincent and Bowler. 2020 Vincent and Bowler This content is distributed under the terms of the Creative Commons Attribution 4.0 International license.

**FIG 3:**
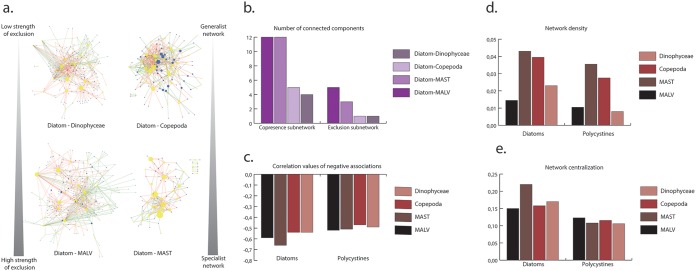
Subnetworks of diatoms with their major interacting groups. (a) The four subnetworks focus on correlations between diatoms and the groups with which they display the highest numbers of associations. Diatom nodes are in yellow, and the corresponding partner (Dinophyceae, Copepoda, MALV, and MAST) nodes are in blue. Green edges correspond to copresences, while red edges correspond to exclusions. The size of the node corresponds to a continuous mapping of its edge number in the global diatom interactome. The gray arrows correspond to ranked network topology values calculated for each network. (b) Number of connected components for each subnetwork, separated by copresence or exclusion (a lower number of connected components suggests stronger connectivity). (c to e) Comparison of exclusion correlation values (c), network density (the ratio between the realized number of edges and the possible number of edges) (d), and network centralization (values closer to 1 indicate a starlike topology) (e) between diatoms and polycystines with their major partners of interaction.

Subnetworks were then extracted for both copresences and exclusions involving diatoms with copepods, dinophyceae, syndiniales (MALVs), and MASTs (a group of small, flagellated, bacterivorous stramenopiles) (Fig. 3a). The size of each node corresponds to a continuous mapping of the betweenness centrality, a number between 0 and 1 that reflects the amount of control (importance) that this node exerts over the interactions of other nodes in the network (48, 49). What is noteworthy is that important diatom genera are not always the same depending on the partner of interaction. More specifically, Syndiniales and Dinophyceae subnetworks involve mainly *Pseudo-nitzschia*-, *Actinocyclus*-, and *Chaetoceros*-assigned barcodes, whereas important nodes in copepods and MASTs involve *Thalassiosira*, *Leptocylindrus*, and *Synedra*, showing a nonrandom pattern of species cooccurrence. Many MAST nodes belong to the MAST-3 clade, known to harbor the diatom parasite Solenicola setigera (50).

In order to compare the architecture between the four subnetworks, we investigated the specificity of the interaction, asking if all organisms are interconnected using topological metrics such as connected components. Diatom-MALV and diatom-MAST subnetworks have more connected components, suggesting more specialist interactions than diatoms with copepods or dinophyceae (Fig. 3b and Table S3), and average scores of exclusions were stronger for diatom-MAST (ρ = −0.66 + −0.09) and diatom-MALV (ρ = −0.59 + −0.09) subnetworks (Fig. 3c).

10.1128/mSystems.00444-19.9 TABLE S3 Network topologies for major diatom genera extracted from the *Tara* Oceans interactome. The most abundant diatom genera were identified based on their read abundance in the *Tara* Oceans metabarcoding data set. Their subnetworks were isolated, and network topologies were computed in Cytoscape. Download Table S3, XLSX file, 0.02 MB. Copyright © 2020 Vincent and Bowler. 2020 Vincent and Bowler This content is distributed under the terms of the Creative Commons Attribution 4.0 International license.

We used polycystines as a comparison group as they were also shown to be segregators. Diatoms have stronger negative scores than polycystines (*t* test *P* value of 5.70 × 10^−10^), reflecting a higher potential as segregators with respect to potential competitors, grazers, and parasites such as copepods, dinophyceae, and syndiniales (Fig. 3c). Furthermore, diatoms tend to form much denser (more interconnected, i.e., less species-specific) (Fig. 3d) and more centralized (relying on fewer central species) (Fig. 3e) networks than polycystines. Despite comparable patterns of segregation between diatoms and polycystines, they differ in the strength of negative interactions based on Spearman correlation values and how specific the interactions are at the barcode level.

### Global-scale genus abundance does not determine importance in connectivity.

While abundant diatoms are likely to be important players in biogeochemical cycles such as NPP and carbon export (51), how their biotic interactions influence plankton community diversity and abundance is still unknown. To address this question, the 10 most abundant diatom genera, defined based on 18S V9 read abundances (27), were analyzed with respect to their positions in the diatom interactome. This analysis revealed that some genera barely play any roles. For example, *Chaetoceros* is the most abundant genus (1,615,027 reads), yet it is represented in only 515 edges across the interactome. Hence, no significant correlation was found between the total abundance of the genus and the number of edges (i.e., putative biotic relations in which the genus is involved) (Spearman *P* value of 0.96) or the number of nodes involved (i.e., the number of different interacting organisms) (Spearman *P* value of 0.45) (Fig. S1). On the other hand, the diatom genus *Synedra*, which is not abundant at the global level (ranked as the 22nd most abundant diatom, with 28,700 reads), was involved in over 100 significant associations. *Pseudo-nitzschia* is the top assigned cooccurring diatom, representing 7% of the positive interactions in the diatom network; on the contrary, exclusions involved a large array of diatom genera, each representing on average 2% of the interactions (Fig. S2).

10.1128/mSystems.00444-19.2 FIG S1 Comparison of total abundances, numbers of interactions, and ratios of positive and negative interactions for the top 10 diatoms. Download FIG S1, PDF file, 0.01 MB. Copyright © 2020 Vincent and Bowler. 2020 Vincent and Bowler This content is distributed under the terms of the Creative Commons Attribution 4.0 International license.

10.1128/mSystems.00444-19.3 FIG S2 Major diatom groups involved in the *Tara* Oceans interactome. A KRONA plot shows the most important diatoms in the *Tara* Oceans interactome based on the taxonomic affiliation of nodes. For example, 2% of the diatom interactions involve mutual exclusion between *Leptocylindrus* and another organism. A total of 81 unique diatom nodes (md5sum) were involved in the interactome. Download FIG S2, PDF file, 0.1 MB. Copyright © 2020 Vincent and Bowler. 2020 Vincent and Bowler This content is distributed under the terms of the Creative Commons Attribution 4.0 International license.

Statistics of network-level properties provide further insights into the overall structure of genus-specific assemblages and were investigated at the genus level for the most connected ones (Table S3). *Leptocylindrus*, *Proboscia*, and *Pseudo-nitzschia* displayed a higher average number of neighbors, meaning that their subnetworks are highly interconnected between diatom and nondiatom OTUs, suggesting that interactions within these genera are not species specific. On the other hand, the *Chaetoceros*, *Eucampia*, and *Thalassiosira* subnetworks displayed larger diameters, meaning that a few diatom OTUs are connected both positively and negatively to a large number of partners that are not connected to any other diatom OTUs, indicative of a more species-specific type of behavior with respect to interactions. No clear correlation was found between the crown age estimation of marine planktonic diatoms or taxon richness estimated from the number of OTU swarms (52) and the number of associations in which they are involved (Table S3), suggesting that the establishment of biotic interactions is a continuous and dynamic process independent of the age of a diatom genus.

### Species-level segregation determined by endemic and blooming diatoms.

**FIG 4:**
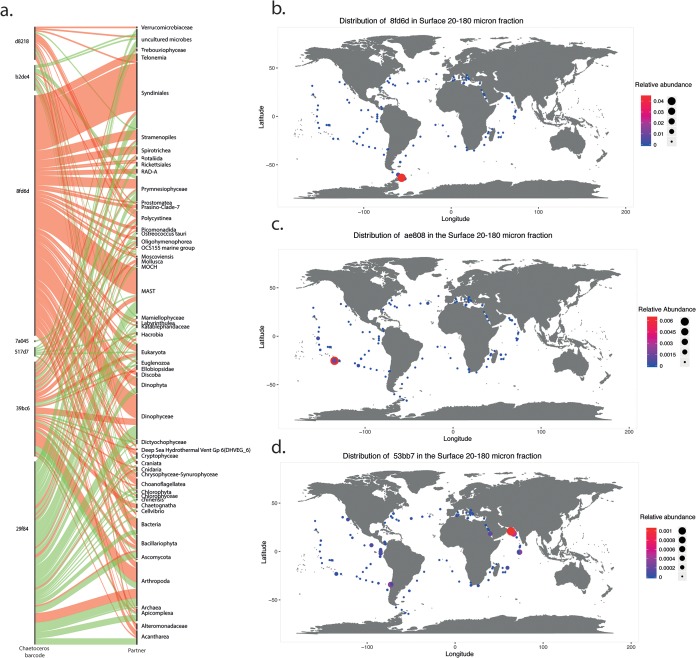
Barcode-level specificity of interactions and biogeographic distribution of the top excluding diatoms. (a) Barcode-level associations of the diatom genus *Chaetoceros*. Diatom barcode annotations are listed on the left, and partners of interaction are on the right. The thickness of the ribbons corresponds to the number of interactions, with copresences in green and exclusions in red. Barcodes 29f84 and 7a045, *Chaetoceros rostratus*; 8fd6d and b2de4 *Chaetoceros* sp.; 39bc6, 517d7, and d8218; Chaetoceros muelleri. (b to d) Biogeography of the top excluding diatom barcodes in surface waters of the 20- to 180-μm size fraction based on the *Tara* Oceans metabarcoding data set (46), 8fd6d (*Chaetoceros rostratus*) (b), ae808 (*Thalassiosira* sp.) (c), and 53bb7 (*Proboscia* sp.) (d).

Due to the small number of individual barcodes in the interactome that have species-level resolution, we decided to conduct a finer analysis and ask whether or not different barcodes of the same (abundant) genera display specificity in the type of interactions and partners with which they interact. We illustrate this barcode specificity with three different examples: *Chaetoceros*, *Pseudo-nitzschia*, and *Thalassiosira*. *Chaetoceros* interactions reveal that different species display very different cooccurrence patterns. Barcode “29f84,” assigned to Chaetoceros rostratus, is essentially involved in copresences, while barcode “8fd6d,” assigned to Chaetoceros debilis, is the major driver of exclusions involving dinophyceae, MASTs, syndiniales, and arthropods (Fig. 4a). This could reflect the different species tolerances to other organisms since several *Chaetoceros* species are known to be harmful to aquaculture industries (53); *Chaetoceros debilis*, in particular, can cause physical damage to fish gills (54).

*Pseudo-nitzschia* barcodes are primarily involved in copresences. However, they display exclusions with organisms such as arthropoda and dinophyceae, and some are known to produce the toxin domoic acid under specific conditions (55). No exclusions regarding syndiniales appear, and barcode-level specificity is observed with barcode “1d16c,” which is involved in a much higher number of interactions than barcode “b56c3.” Unfortunately, these diatom sequences were not assigned at the species level. Finally, the *Thalassiosira* subnetwork displays mostly exclusions with syndiniales, arthropoda, and polycystines, with one of the three representative barcodes (“53bb7”) being responsible for 93% of the exclusions (Table S3).

The distribution of the above-mentioned diatoms, involved in a high number of mutual exclusions, is typical of that of endemic and blooming diatoms, as their read abundance massively increases in either specific localized stations or nutrient-replete well-mixed regions. This observation was supported by analyzing the distribution patterns of the 6 top diatom barcodes involved in exclusions (Table S3), such as barcodes 90dad (226 exclusions; unassigned *Bacillariophyta* blooming in Indian Ocean station TARA_036), 4c4a8 (193 exclusions; *Raphid-Pennate*, Marquesas station TARA_122), 8fd6d (168 exclusions; *Chaetoceros* in Southern Ocean station TARA_088) (Fig. 4b), 30191 (166 exclusions; *Actinocyclus* in Indian Ocean station TARA_033), 53bb72 (94 exclusions; *Thalassiosira* in Indian Ocean station TARA_036) (Fig. 4c), and ae808 (103 exclusions; *Proboscia* in station TARA_116) (Fig. 4d).

### Diatom-bacterium interactions in the open ocean.

**FIG 5:**
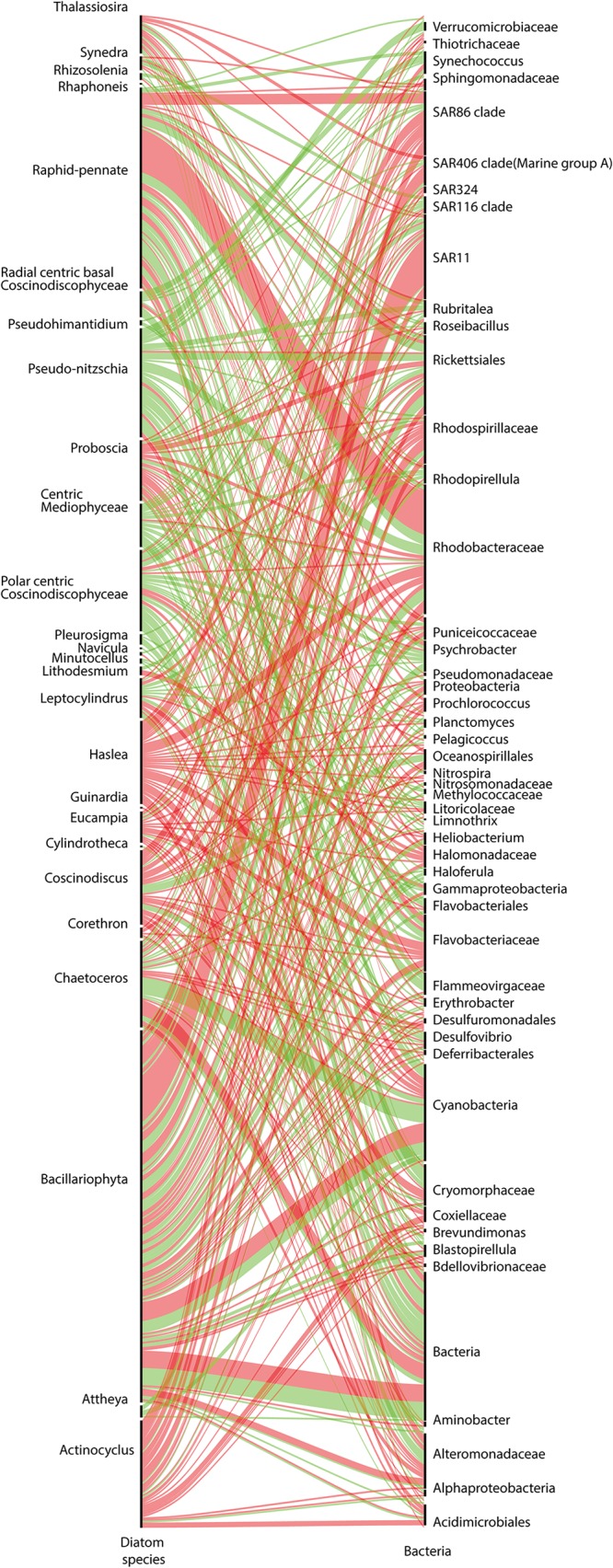
Diatom-bacterium interactions. Diatom taxonomic annotations are listed on the left, and partners of interaction are on the right. The thickness of the ribbons corresponds to the number of interactions, with copresences in green and exclusions in red.

Diatom-prokaryote associations represent 830 interactions, or 19% of the whole diatom cooccurrence network. This can be considered average compared to bacterial associations in copepod interactions (28%), dinophyceae (18.5%), radiolaria (20.5%), and syndiniales (16.3%). By classifying the bacteria according to their primary nutritional group (see Materials and Methods), diatoms were found to be more associated, both positively and negatively, with heterotrophs (637 associations) than with autotrophs (87 associations) (Fig. S3). Even though diatoms do not significantly cooccur with or exclude a specific bacterial nutritional group, many exclusions involve *Rhodobacteraceae* and the SAR11 and SAR86 clades (Fig. 5). Interestingly, diatom-specific patterns are apparent. For example, the *Actinocyclus* and *Haslea* diatom genera are solely involved in exclusions against a wide range of bacteria, whereas *Pseudo-nitzschia* is mainly involved in copresences. Interestingly, Haslea ostrearia is known for producing a water-soluble blue pigment, marennine, against which closely related pigments display antibacterial activities (56).

10.1128/mSystems.00444-19.4 FIG S3 Distribution of diatom-bacterium associations in the open ocean. Diatom-bacterium interactions were derived from the global interactome. Bacteria involved are listed in abscissa and colored by trophic mode (black, autotrophs; red, heterotrophs; blue, unknown). The partner of the interaction in which they are involved is represented by the colored bars. Download FIG S3, PDF file, 0.1 MB. Copyright © 2020 Vincent and Bowler. 2020 Vincent and Bowler This content is distributed under the terms of the Creative Commons Attribution 4.0 International license.

### Skewed knowledge about diatom biotic associations.

**FIG 6:**
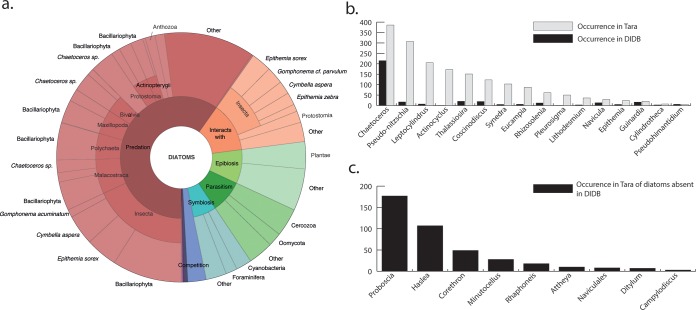
Current knowledge of diatom biotic interactions and comparison with the *Tara* Oceans interactome. (a) KRONA plot based on available literature concerning diatom associations mined and manually curated from Web of Science, PubMed, and Globi and made available online (https://doi.org/10.5281/zenodo.2619533). The outer circle represents the diatom genera (when known), the middle circle represents the interacting partner, and the inner circle represents the type of interaction (predation, parasitism, or symbiosis). (b) Comparison between the numbers of interactions involving a specific diatom genus in the literature (black) and in the *Tara* Oceans interactome (gray) showing strong disparities for diatoms such as *Pseudo-nitzschia.* (c) Numbers of interactions of important diatom genera in the interactome that are absent from the literature, suggesting interesting areas for future research.

To review current knowledge about diatom interactions, we generated an online open-access database (https://doi.org/10.5281/zenodo.2619533) that assembled the queryable knowledge in the literature about diatom associations from both marine and freshwater habitats and is synchronized with Globi, a global effort to map biological interactions (43). It contains a total of 1,533 associations from over 500 papers involving 83 genera of diatoms and 588 genera of other partners, illustrating a diversity of association types, such as predation, symbiosis, allelopathy, parasitism, and epibiosis, as well as a diversity of partners involved in the associations, including both prokaryotes and eukaryotes and micro- and macroorganisms (Fig. 6a). However, despite our systematic effort, it is unlikely that we captured everything.

We noted that 58% (883 out of 1,533) of the interactions are labeled “eatenBy” (“Predation” in Fig. 6a) and involve mainly insects (267 interactions; 30% of diatom predators) and crustaceans (15% of diatom predators). Cases of epibiosis, representing approximately 10% of the literature database, were largely dominated by epiphytic diatoms living on plants (40% of epibionts) and epizoic diatoms living on copepods (9% of epibionts). Parasitic and photosymbiotic interactions, although known to have significant ecological implications on the individual-host level as well as on a community composition scale (57), represented only 15% of the literature database, for a total of 219 interactions, involving principally diatom associations with radiolarians and cyanobacteria. Interactions involving bacteria represent 72 associations (4.8% of the literature database).

The distribution of habitats among the studied diatoms reveals a singular pattern: the majority of diatom interactions in the literature are represented by a few freshwater diatoms, whereas many marine species are reported in just a small number of interactions (Fig. S4). In terms of partners involved (detailed in Fig. S5), one-third are represented by insects feeding upon diatoms in streams and crustaceans feeding upon diatoms in both marine and freshwater environments. Other principal partners are plants, upon which diatoms attach as “epiphytes,” such as *Posidonia* (seagrass), *Potamogeton* (pondweed), *Ruppia* (ditch grass) and *Thalassia* (seagrass). Consequently, our knowledge based on the literature produces a highly centralized network containing a few diatoms mainly subject to grazing or epiphytic on macroorganisms. Major diatom genera for which interactions are reported in the literature are *Chaetoceros* spp. (215 interactions; marine and freshwater), Epithemia sorex (135 interactions; freshwater), and Cymbella aspera (115 interactions; freshwater).

10.1128/mSystems.00444-19.5 FIG S4 Habitats of diatoms involved in known interactions. For each available diatom genus in the literature interaction database, habitat was assigned based on available knowledge. Download FIG S4, PDF file, 0.1 MB. Copyright © 2020 Vincent and Bowler. 2020 Vincent and Bowler This content is distributed under the terms of the Creative Commons Attribution 4.0 International license.

10.1128/mSystems.00444-19.6 FIG S5 Main partners involved in diatom interactions based on the literature. Download FIG S5, PDF file, 0.1 MB. Copyright © 2020 Vincent and Bowler. 2020 Vincent and Bowler This content is distributed under the terms of the Creative Commons Attribution 4.0 International license.

### Overlapping empirical evidence from data-driven results reveals gaps in knowledge.

In an effort to improve edge annotation in the cooccurrence network, the literature database presented here was used. The occurrence of a specific genus in the literature was compared to its occurrence in the *Tara* Oceans interactome. On average, the cooccurrence network revealed many more potential links between species than what has been reported in the literature (Fig. 6b). Disparity was especially high for *Pseudo-nitzschia*, mentioned in 17 interactions in the literature compared to 307 associations in the interactome. On the other hand, many diatoms involved in several associations in the interactome are absent from the literature, such as *Proboscia* and *Haslea* (Fig. 6c).

Of 1,533 literature-based interactions, 178 could potentially be found in the *Tara* Oceans interactome, as both partners had a representative barcode in the *Tara* Oceans database. A total of 33 literature-based interactions (18.5% of the literature associations) were recovered in the network at the genus level, representing a total of 289 interactions from the interactome and 209 different barcodes. These 289 interactions represent 6.5% of all the associations involving Bacillariophyta in the *Tara* Oceans cooccurrence network. By mapping available literature on the cooccurrence network, we can see that the major interactions recovered are those involving competition, predation, and symbiosis with arthropods, dinoflagellates, and bacteria. However, predation by polychaetes and parasitism by cercozoa and chytrids are missing from the *Tara* Oceans interactome.

## DISCUSSION

The *Tara* Oceans interactome represents an ideal case study to investigate global-scale community structure involving diatoms, as it maximizes spatiotemporal variance across a global sampling campaign and captures systems-level properties. Here, we reveal that diatoms and polycystines are the organismal groups with the highest proportions of exclusions within the *Tara* Oceans interactome and classify them as segregators according to a definition described previously (47), as they display more negative than positive associations. Diatoms and polycystines prevent their cooccurrence with a range of potentially harmful organisms over broad spatial scales (Fig. 1a and d), a pattern unseen in the other photosynthetic classes examined (Fig. 1b and c), reflected by a significant exclusion of major functional groups of predators, parasites, and competitors such as copepods, Syndiniales, and Dinophyceae (Fig. 1e).

Diatoms are known to have developed an effective arsenal composed of silicified cell walls, spines, toxic oxylipins, and chain formation to increase size, so we propose that the observed exclusion pattern reflects the worldwide impact of the diatom arms race against potential competitors, grazers, and parasites. Additionally, building upon the phylogenetic affiliation of individual sequences, barcodes can be assigned to a plankton functional type that refers to traits such as the trophic strategy and role in biogeochemical cycles (58). As demonstrated in the *Tara* Oceans interactome (46), diatoms compose the “phytoplankton silicifiers” metanode and display a variety of mutual exclusions that again distinguish them from other phytoplankton groups. The role of biotic interactions is emphasized by the fact that out of the complete diatom association network, colocalization and coexclusion of diatoms with other organisms are due to shared preferences for an environmental niche in 13% of the cases, emphasizing the importance of biotic factors in 87% of the associations (Fig. 2).

Diatom-MAST and diatom-MALV networks display more specialist interactions than diatom-copepod and diatom-Dinophyceae networks (Fig. 3b). Correlation values reveal stronger exclusion patterns of diatoms against MASTs and MALVs (Fig. 3c). These properties are conserved in the other segregator group, polycystines. Yet diatoms outcompete polycystines with higher strengths of exclusions based on correlation values and denser networks suggesting more species-specific interactions in polycystines (Fig. 3c to e). Previous work exploring abundance patterns among planktonic silicifiers in the *Tara* Oceans data (26) revealed strong size-fractionated communities: while the smallest-sized fraction (0.8 to 5 μm) contained a large diversity of silicifying organisms in nearly constant proportions, cooccurrence of diatoms and polycystines was rare in larger-sized fractions (20 to 180 μm), where the presence of one organism appeared to exclude the presence of the other.

Analysis at the genus level shows that abundant diatoms such as *Attheya* do not prevail in the network, contrary to *Synedra*, which, on a global scale, is less significant in terms of abundance but is highly connected to the plankton community. We show the existence of a species-level segregation effect that can be attributed to harmful traits (54) (Fig. 4a), reflected by blooming and endemic distribution patterns for the top segregating diatoms (Fig. 4b to d). These results support previously reported observations indicating the importance of biotic interactions in affecting ocean planktonic blooms and distribution (29, 59). However, we cannot discount environmental parameters, as diatom blooms are also known to be triggered by light and nutrient perturbation.

Our literature survey reveals a skewed knowledge, focusing on freshwater diatoms and interactions with macroorganisms, with very few parasitic, photosymbiotic, or bacterial associations (Fig. 6a). The relative paucity of marine microbial studies can be explained by the difficulty in accessing these interactions in the field, which obviously limits our understanding of how such interactions structure the community on a global scale. Comparing empirical knowledge and data-driven association networks reveals understudied genera, such as *Leptocylindrus* and *Actinocyclus*, and those that are not even present in the literature, such as *Proboscia* and *Haslea* (Fig. 6b and c). However, *Proboscia* is a homotypic synonym of *Rhizosolenia* that is found in the interactome, which illustrates the consequences of nonuniversal taxonomic denominations on diversity analysis.

While 18.5% of the literature database was recovered in the interactome, it explained only 6.5% of the 4,369 edges composing the diatom network. The gap between the 20% of diatom-bacterium interactions in the *Tara* Oceans interactome and only 4.8% of diatom-bacterium associations described in the literature highlights how little we know about host-associated microbiomes at this time. Most of the experimental studies focus on symbiosis with diazotrophs (16) and dinoflagellates (60) and the antibacterial activity of *Skeletonema* against bacterial pathogens (61). In many ways, this high proportion of unmatched interactions should be regarded as the “unknown” proportion of microbial diversity emerging from metabarcoding surveys. Part of it is truly unknown and new, part of it is due to biases in data gathering and processing, and part of it is due to the lack of an extensive reference database. Indeed, the current literature is biased toward model organisms and species that can be easily cultured as well as diatoms with biotechnological potential.

This study faces challenges regarding the computation, analysis, and interpretation of cooccurrence networks while suggesting their potential to uncover processes governing diatom-related microbial communities. Further studies should compare diatom networks using several cooccurrence methods (62), taxonomic levels (63), and theoretical frameworks (47, 64, 65). Assigning biological interactions such as predation, parasitism, or symbiosis to correlations will require enhanced references of biotic interactions (34), of which the open-source collaborative database provided in this paper is an addition that also highlights potential research avenues. Furthermore, a vast body of literature already exists in the field of ecological networks, traditionally focusing on observational noninferred data and the modeling of food webs and host-parasite and plant-pollinator networks (66, 67). Various properties linked to the architecture of these antagonistic and mutualistic networks have been formalized, such as nestedness, modularity, or the impact of combining several types of interactions in a single framework (68, 69). These works have inspired this study, and we envision that enhanced cross-fertilization between the disciplines of ecological networks and cooccurrence networks would highly benefit both communities, ultimately helping to understand the laws governing the “tangled bank” (70).

Diatoms have undoubtedly succeeded in adapting to the ocean’s fluctuating environment, shown by recurrent, predictable, and highly diverse bloom episodes (71). They are considered r-selected species with high growth rates under favorable conditions that range from nutrient-rich highly turbulent environments to stratified oligotrophic waters (24, 72, 73). Their success has long been attributed to this ecological strategy; here, we suggest that abiotic factors alone are not sufficient to explain their ecological success. The present study shows that diatoms do not cooccur with potentially harmful organisms such as predators, parasites, and pathogens (74), shedding light on the top-down forces that could drive diatom evolution and adaptation in the modern ocean.

## MATERIALS AND METHODS

### Relative proportions of cooccurrences and exclusions with respect to major partners and network analysis.

All analyses were performed on a cooccurrence network reported previously (46). Environmental drivers of diatom-related edges are shown in Fig. 2. Four independent matrices were created from the interactome regarding the major partners interacting with diatoms (copepods, dinophyceae, syndiniales, and radiolaria), containing only pairwise interactions that involved the major partner, and binomial testing was done using the dbinom and pbinom functions as implemented in the stats package of R version 3.3.0. Subnetwork topologies were analyzed using the NetworkAnalyzer plug-in in Cytoscape (75), as described previously (76).

### Major diatom interactions.

The 10 most abundant diatom genera in the surface ocean were selected based on work reported previously (27). Their cooccurrence network was extracted from the global interactome and analyzed at the ribotype level. Network topologies are available in Table S3 in the supplemental material. The distribution of individual barcodes was assessed across the 126 *Tara* Oceans sampling stations.

### Construction of the diatom interaction literature database.

Literature was screened up to November 2017 to look for all ecological interactions involving diatoms to establish the current state of knowledge regarding the diatom interactome, in both marine and freshwater environments (available at https://doi.org/10.5281/zenodo.2619533). It is designed to be completed by external contributions. Diatom ecological interactions as defined in this paper are a very large group of associations, characterized by (i) the nature of the association defined by the ecological interaction or the mechanism (predation, symbiosis, mutualism, competition, or epibiosis), (ii) the diatom involved, and (iii) the partners of the interaction.

The protocol to build the list of literature-based interactions was as follows: (i) collect publications involving diatom associations using (a) the Web of Science query TITLE: (diatom*) AND TOPIC: (symbio* OR competition OR parasit* OR predat* OR epiphyte OR allelopathy OR epibiont OR mutualism), (b) Eutils tools to mine PubMed and extract identifications of all publications with the search URL https://eutils.ncbi.nlm.nih.gov/entrez/eutils/esearch.fcgi?db=pubmed&term=diatom+symbiosis&usehistory=y and the same keywords, (c) the get_interactions_by_taxa(sourcetaxon = “Bacillariophyta”) function from the RGlobi package (43), the most recent and extensive automated database of biotic interactions, and (d) personal mining from other publication browsers and input from experts; (ii) extract, when relevant, the partners of the interactions based on the title and on the abstract for Web of Science, PubMed, and personal references and normalize the label of the interaction based on Globi nomenclature; and (iii) display a KRONA plot with Type of Interaction/Partner Class/Diatom genus/Partner genus_species (Fig. 6a). Cases of epipsammic (sand) and epipelic (mud) interactions were not considered, as they involved associations with nonliving surfaces.

### Comparison of literature interactions and the diatom interactome.

All partner genera interacting with diatoms based on the literature were searched for in the *Tara* Oceans data set based on the lineage of the barcode. For each barcode that had a match, identifiers (“md5sum”) were extracted, creating a list of 954,110 barcodes to be searched for in the global interactome.
