# Supplementary figures and images for: Diatoms Are Selective Segregators in Global Ocean Planktonic Communities
Source: mSystems. 2020 Jan 21;5(1):e00444-19. doi: 10.1128/mSystems.00444-19 (PMC6977069; doi:10.1128/mSystems.00444-19)

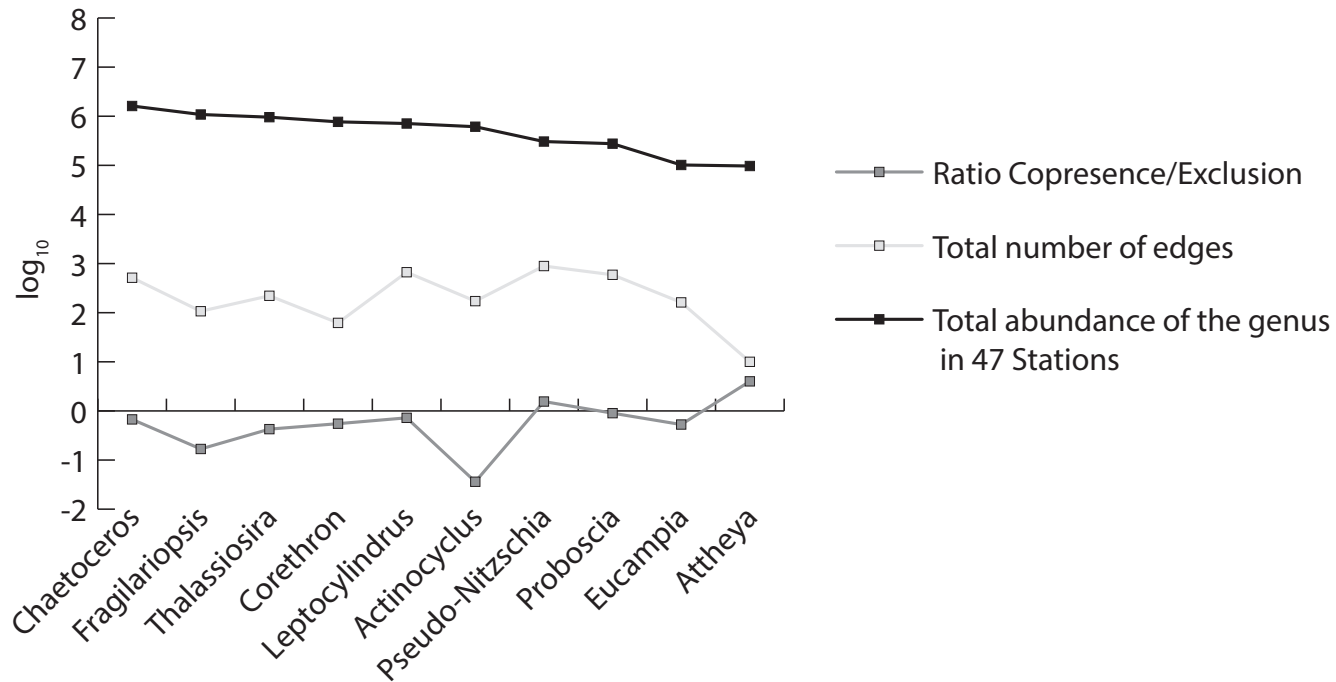

Supplement: FIG S1 [file mSystems.00444-19-sf001.pdf]

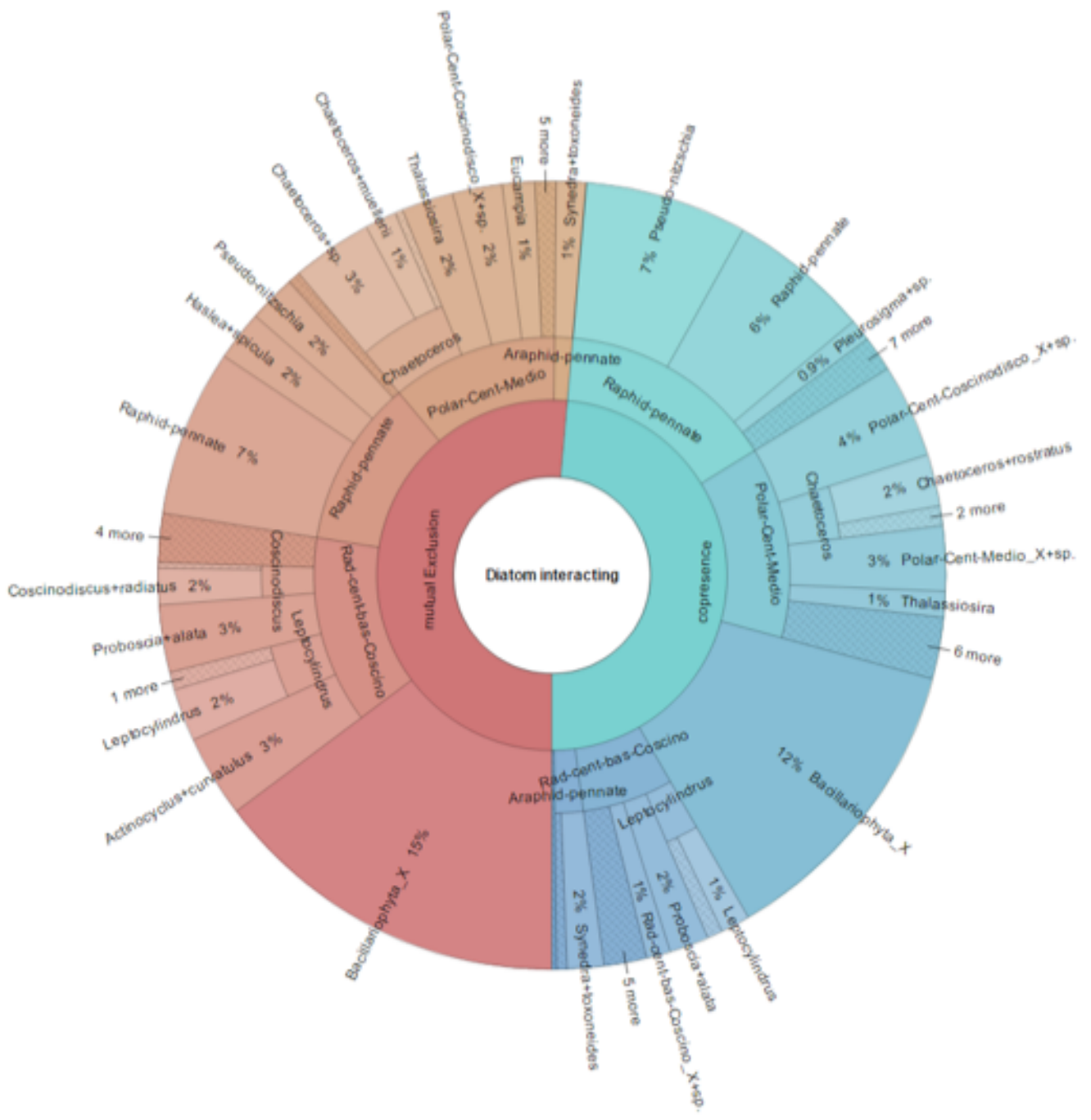

Supplement: FIG S2 [file mSystems.00444-19-sf002.pdf]

Habitats of known diatoms interactions

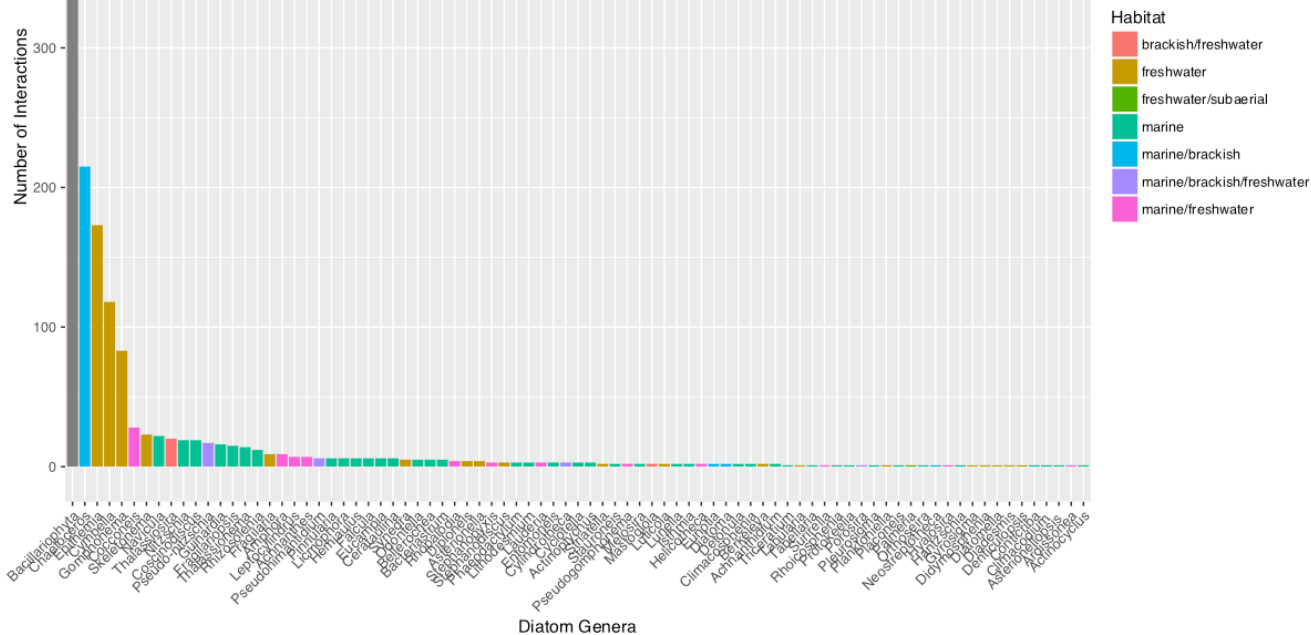

Supplement: FIG S4 [file mSystems.00444-19-sf004.pdf]

### Main partners involved

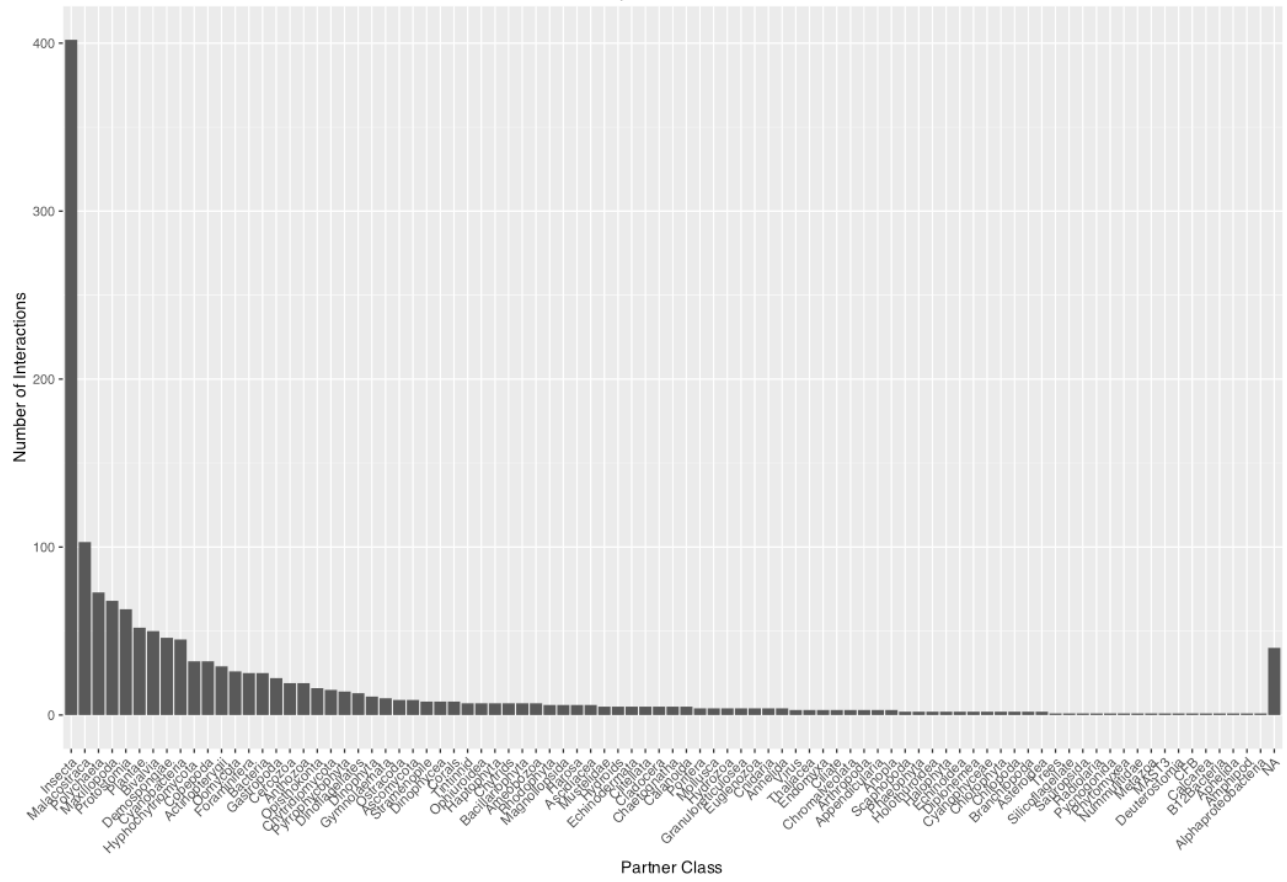

Supplement: FIG S5 [file mSystems.00444-19-sf005.pdf]
