# Supplementary material for: Diatoms Are Selective Segregators in Global Ocean Planktonic Communities
Source: mSystems. 2020 Jan 21;5(1):e00444-19. doi: 10.1128/mSystems.00444-19 (PMC6977069; doi:10.1128/mSystems.00444-19)
Supplement: FIG S3 [file mSystems.00444-19-sf003.pdf]

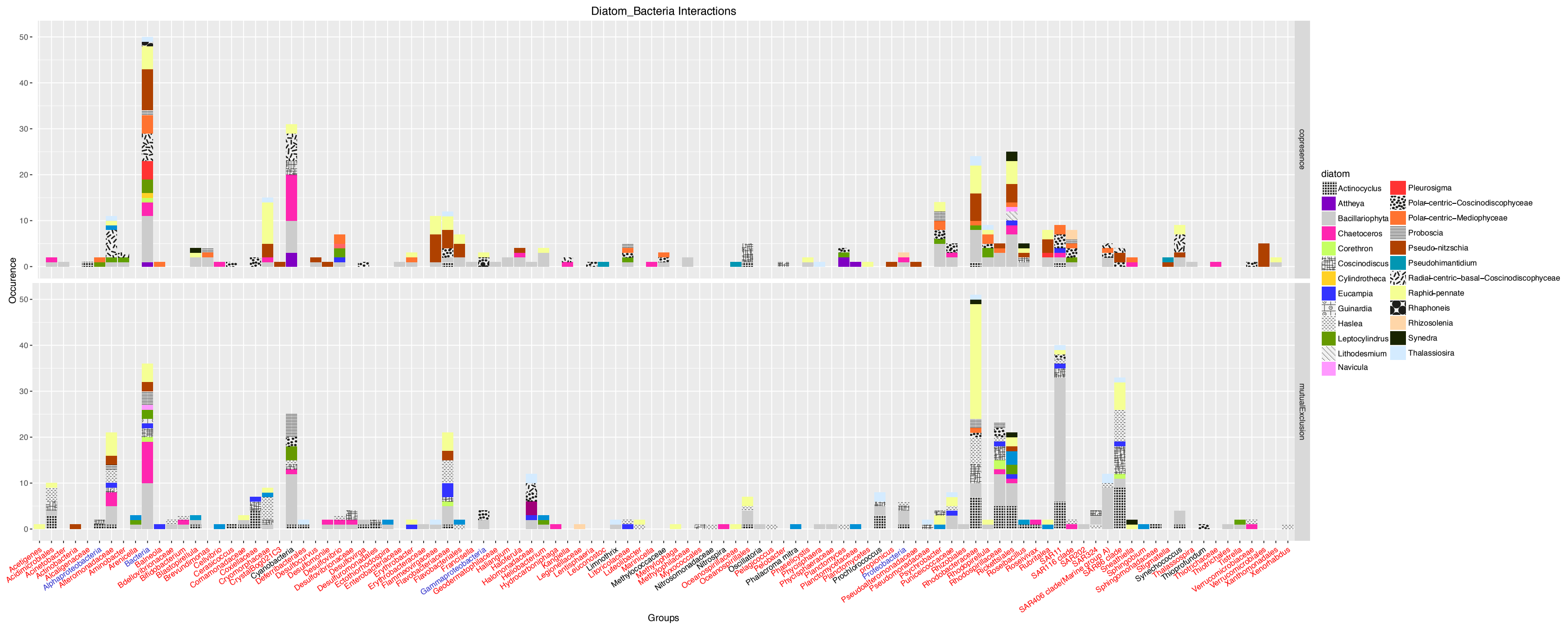

Diatom_Bacteria Interactions
copresence
mutualExclusion
Occurence
Groups
diatom
Actinocyclus
Attheya
Bacillariophyta
Chaetoceros
Corethron
Coscinodiscus
Cylindrotheca
Eucampia
Guinardia
Haslea
Leptocylindrus
Lithodesmium
Navicula
Pleurosigma
Polar–centric–Coscinodiscophyceae
Polar–centric–Mediophyceae
Proboscia
Pseudo–nitzschia
Pseudohimantidium
Radial–centric–basal–Coscinodiscophyceae
Raphid–pennate
Rhaphoneis
Rhizosolenia
Synedra
Thalassiosira
Acetigenes
Acidimicrobiales
Acinetobacter
Actinobacteria
Alcaligenaceae
Alphaproteobacteria
Alteromonadaceae
Aminobacter
Arenicella
Bacteria
Balneola
Bdellovibrionaceae
Bifidobacterium
Blastopirellula
Brevundimonas
Cellvibrio
Cerasicoccus
Comamonadaceae
Coxiellaceae
Cryomorphaceae
CrystalBog021C3
Cyanobacteria
Deferribacterales
Desulfocurvus
Desulfomonile
Desulfovibrio
Desulfovibrionaceae
Desulfovirga
Desulfuromonadales
Ectothiorhodospira
Enterobacteriaceae
Erythrobacter
Erythrobacteraceae
Flammeovirgaceae
Flavobacteriaceae
Flavobacteriales
Francisella
Gammaproteobacteria
Geodermatophilaceae
Haliangium
Haloferula
Halomonadaceae
Heliobacterium
Hydrocarboniphaga
Kangiella
Legionellaceae
Lentisphaeria
Leuconostoc
Limnothrix
Litoricolaceae
Luteolibacter
Marinicella
Methylococcaceae
Methylophaga
Methylophilaceae
Myxococcales
Nitrosomonadaceae
Nitrospira
Oceanospirillaceae
Oceanospirillales
Oscillatoria
Pelagicoccus
Pelobacter
Phalacroma mitra
Phaselicystis
Phycisphaera
Phycisphaeraceae
Planctomyces
Planctomycetaceae
Planctomycetes
Prochlorococcus
propionicus
Proteobacteria
Pseudoalteromonadaceae
Pseudomonadaceae
Psychrobacter
Puniceicoccaceae
Rhizobiales
Rhodobacteraceae
Rhodopirellula
Rhodospirillaceae
Rickettsiales
Roseibacillus
Roseivivax
Rubritalea
SAR11
SAR116 clade
SAR202
SAR324
SAR406 clade(Marine group A)
SAR86 clade
Sneathiella
Sphingobium
Sphingomonadaceae
Stigmatella
Synechococcus
Thalassospira
Thioprofundum
Thiotrichaceae
Thiotrichales
Tistrella
Verrucomicrobiaceae
Verrucomicrobiales
Xanthomonadales
Xenorhabdus
